# Cognitive therapies and their impact on neuropsychiatric symptoms in mild cognitive impairment or dementia: a scoping review

**DOI:** 10.3389/fpsyg.2025.1547619

**Published:** 2025-04-10

**Authors:** Beatriz Lara-Consuegra, Anna Carnes-Vendrell, Paula Torres-Hidalgo, Gerard Piñol-Ripoll

**Affiliations:** ^1^Unitat Trastorns Cognitius, Clinical Neuroscience Research, Hospital Universitari Santa Maria, IRBLleida, Lleida, Spain; ^2^NeuroLleida, ASPID’s Neurorehabilitation Service, Lleida, Spain

**Keywords:** mild cognitive impairment, dementia, neuropsychiatric symptoms, nonpharmacological treatment, cognitive therapy

## Abstract

To date, the impact of cognitive therapies on patients with dementia and mild cognitive impairment (MCI) has been studied in terms of cognition, quality of life and activities of daily living rather than in the context of neuropsychiatric symptoms (NPS). The objective of this scoping review was to collect evidence that different cognitive therapies affect the NPS of patients with MCI and dementia. A bibliographic search was conducted in the PUBMED, EMBASE, ISI WOS and SCOPUS databases through February 2025. After the elimination of duplicates, a total of 1,854 publications were identified. Among these, 42 articles were included in the analysis. These articles were reviewed by title and abstract, and then the full text was reviewed by two independent researchers with subsequent decisions about conflicts made in consultation with a third researcher. A sample of 4,089 participants was collected. All participants had undergone cognitive training interventions, cognitive rehabilitation, cognitive stimulation, art therapy, reminiscence therapy or psychobehavioural therapy and were evaluated for NPS. Depression was the most commonly analysed symptom (70% of the articles). The types of interventions that improved NPS the most were art therapy and reminiscence therapy (76 and 71% of the articles), whereas cognitive training (43%) was the least effective. While all types of therapy seemed to support the improvement of NPS, art therapy and reminiscence therapy were the most effective, especially for patients with MCI or dementia. However, studies that comprehensively evaluate the effects of cognitive therapy on NPS other than depression are lacking.

## 1 Introduction

Alzheimer’s disease (AD) is the primary cause of dementia in geriatric patients (Dumurgier and Tzourio, 2020), and mild cognitive impairment (MCI) is a prodromal stage of AD (Albert et al., 2011). Increased longevity is causing a worldwide pandemic of MCI, AD, and AD-related dementias (ADRD; Atri, 2019). Currently, it is estimated that MCI has a prevalence of approximately 18.9% (Petersen et al., 2014); however, dementia is twice as prevalent as MCI (Sánchez Rodríguez and Torrellas, 2011). Although it is difficult to determine the probability of progression, studies of clinical cohorts of patients with MCI have reported that the annual rate of progression to dementia ranges from 10 to 15% (Farias et al., 2009). To combat this progression, the reduction of risk factors and the promotion of intervention strategies are essential (Shimada et al., 2019).

Studies have shown that cognitive exercise has a small effect on the quality of life (QoL) and activities of daily living (ADL) of people with mild to moderate dementia with no negative effects reported at any level (Woods et al., 2023). In contrast to the evidence collected on cognitive functioning, much less is known about cognitive therapy and its impact on neuropsychiatric symptoms (NPS), which are noncognitive symptoms that are behavioural or psychological in nature and are associated with cognitive impairment due to MCI or dementia (Lyketsos et al., 2011).

Research has shown that moderate to advanced stages of dementia are associated with NPS (Fischer and Agüera-Ortiz, 2017). However, it is also important to note the presence of NPS in patients with MCI because NPS may be a predictor of worse prognosis; furthermore, NPS are related to the progression of MCI to dementia (Palmer et al., 2007). In fact, NPS, especially mood and anxiety symptoms, may occur in prodromal dementia, and symptoms of psychosis may be associated with mortality and progression to dementia (Fischer and Agüera-Ortiz, 2017).

Most NPS are best managed with personalised nonpharmacological approaches, except for symptoms that pose a risk to others, such as aggression or psychosis, for which pharmacological treatment may be the best option (Loi et al., 2018).

Cognitive exercises can be classified into three groups: cognitive training (CT), cognitive rehabilitation (CR) and cognitive stimulation (CS; García-Ribas et al., 2023). CT focuses on cognitive processes that can improve various domains of cognition (García-Ribas et al., 2023) and involves guided practice with a set of standard tasks (Clare and Woods, 2004). CR is a personalised cognitive intervention with specific cognitive targets that aims to enhance patients’ functionality (García-Ribas et al., 2023; Clare and Woods, 2004). CS is a more general approach that includes activities involving cognitive processes and is often conducted in a social context (García-Ribas et al., 2023; Clare and Woods, 2004). Art-based interventions are another type of cognitive treatment to maintain the cognitive health of older adults or even halt the progression of dementia (Masika et al., 2020; Lee et al., 2019). Art therapy includes various forms, such as music, visual art and drama therapy (Fong et al., 2020). Another type of cognitive treatment is reminiscence therapy, which involves sharing and discussing past life events, activities and experiences individually or in a group, usually with the aid of tangible prompts (Pittiglio, 2000; Spector et al., 2000). It is primarily used to improve mood and cognition (Thomas and Sezgin, 2021).

Several studies have investigated how cognitive therapies affect the cognitive function of older people, including cognitively healthy adults and patients with MCI or dementia (Gómez-Soria et al., 2023b; Carrion et al., 2018). Combined cognitive and physical therapies have been employed for patients with MCI or dementia (Karssemeijer et al., 2017). However, it remains unclear how these therapies impact NPS. To the best of our knowledge, only one systematic review has shown an effect of nonpharmacological therapies (including cognitive therapies) on NPS that resulted in decreased levels of agitation (Livingston et al., 2014).

Why might we believe that cognitive therapies can improve NPS? We consider two possible hypotheses. The first is a neurobiological explanation. Studies have shown that neurobiological changes in the brain, particularly in patients with neurodegenerative dementias such as AD, are linked to neuropsychiatric symptomatology (Holthoff et al., 2005; Benoit et al., 2004; Loose et al., 2003; McPherson et al., 2002; Nakaaki et al., 2007; Huang et al., 2024; Baudic et al., 2006; Alvarez and Emory, 2006). For example, AD patients with apathy symptoms present with hypometabolism in orbitofrontal regions. Both the prefrontal cortex and the anterior cingulate gyrus, which are responsible for executive functions, have been implicated, with hypoperfusion observed in the anterior cingulate gyrus and dorsolateral prefrontal regions in patients with depressive symptomatology (Holthoff et al., 2005; Benoit et al., 2004). Cognitive therapies that target executive functions such as working memory, flexibility, reasoning, and abstract thinking (which are particularly affected in prodromal and mild AD) have been shown to increase regional cerebral blood flow and metabolism in the frontal lobe. This area also controls behavioural symptoms such as apathy and depression as well as executive functions such as inhibitory control, which, when impaired, may lead to disinhibition, euphoria, or appetite disorders (Baudic et al., 2006; Alvarez and Emory, 2006). In addition to brain changes, another theory suggests that depression in AD patients may arise as a reaction to cognitive decline. Therefore, the second hypothesis concerning why cognitive therapies impact NPS is that experiencing successful and positive events through cognitive treatment may improve self-confidence and psychological well-being, which may be reflected in NPS assessments such as the NPI (Balestrieri, 2000).

After reviewing the previous literature, we aimed to explore the impact of cognitive therapies on NPS in people with MCI or dementia. The aims of this present scoping review were (i) to identify the types of available evidence on the efficacy of cognitive treatments for NPS in MCI or neurodegenerative dementia, (ii) to examine how research is conducted in this field, and (iii) to identify and discuss the knowledge gaps.

## 2 Methods

A scoping review methodology was used to address the research aims. Scoping reviews provide an excellent opportunity to investigate broad and new topic areas, and they are an appropriate method for including different study designs (Arksey and O’Malley, 2005). The Preferred Reporting Items for Systematic Reviews and Meta-Analyses extension for Scoping Reviews (PRISMA-ScR2018) reporting guidelines were followed throughout this review (Tricco et al., 2018).

The eligibility criteria were as follows.

Inclusion criteria:

- Cognitive disorders: MCI, AD or dementia.
- Nonpharmacological cognitive treatments: cognitive stimulation, cognitive training, reminiscence therapy, and art-based interventions.
- Assessment of objective measures of neuropsychiatric symptoms.
- Articles published from January 2013 to February 2025.

Exclusion criteria:

- Intellectual disabilities.
- Psychiatric diseases.
- Parkinson’s disease or vascular cognitive impairment.
- Online therapies, web-based therapies, computerised therapies.
- Combined exercise and cognitive programmes.
- Reviews.
- Qualitative studies.
- Studies published in languages other than English, Spanish or Catalan.

Relevant studies were identified from January 2013 to February 2025 in the PUBMED, EMBASE, ISI WOS, and SCOPUS databases. The search strategy was adjusted for use in each database, and MESH terms, Boolean operators, truncations and search limits were used (Table 1). Mendeley, a reference manager software, was used throughout the study selection process. Duplicates were removed, and screening was completed via Mendeley software. The title screening was conducted by one researcher (BL-C), while the abstract and full-text screening was conducted by two independent researchers (BL-C and PT-H) who were blinded to the other researchers’ decisions. Conflicting decisions were resolved by a third researcher (AC-V). As recommended by Levac and colleagues, an iterative process was employed for the study selection stage (Levac et al., 2010).

**Table 1:** Database search results.

Database	N	Search Strategy 12/02/2025
Pubmed*	1,336	(((“Alzheimer Disease”[Mesh] OR “Alzheimer Disease”[tiab] OR “Cognitive Dysfunction”[Mesh] OR “Cognitive Dysfunction”[tiab] OR “Neurocognitive Disorders”[Mesh] OR “Cognitive Dysfunction”[tiab] OR “Cognition Disorders”[Mesh]) OR “Dementia”[Mesh] OR “Dementia”[tiab] OR “Mild cognitive impairment”[tiab] NOT “Schizophrenia”[Mesh] NOT “Schizophrenia”[tiab] NOT “Depressive Disorder, Major”[Mesh] NOT “Major Depressive Disorder”[tiab] NOT “Bipolar Disorder”[Mesh] NOT “Bipolar Disorder”[tiab] NOT “Vascular Diseases”[Mesh] NOT “Parkinsonian Disorders”[Mesh] NOT “Parkinson’s Disease”[tiab] NOT “Parkinson Disease, Secondary”[Mesh]) AND (“Neurobehavioural Manifestations”[Mesh] OR “Mood”[tiab] OR “Behavioural Symptoms”[Mesh]”) AND (“Cognitive Behavioural Therapy”[Mesh] OR “Cognitive Behavioural Therapy”[tiab] OR “Cognitive Stimulation [tiab] OR “Cognitive Training”[tiab] OR “Game Training”[tiab] OR “Cognitive Dysfunction/rehabilitation”[Mesh] OR “Cognitive Dysfunction/therapy”[Mesh] OR “Amnesia/rehabilitation”[Mesh] OR “Amnesia/therapy”[Mesh] OR “Games, Experimental”[Mesh] OR “Games, Recreational”[Mesh] NOT “Video Games”[Mesh] NOT “Video Games”[tiab] NOT “Mindfulness”[Mesh] NOT (“Mindfulness”[tiab] NOT “Virtual Reality”[Mesh] NOT “Virtual Reality”[tiab] NOT “Virtual Reality Exposure Therapy”[Mesh] NOT “Exergaming”[Mesh] NOT “Exergaming”[tiab])))
Web of Science*	325	(((((((((TS = (Alzheimer)) OR TS = (cognitive dysfunction)) OR TS = (neurocognitive disorder)) OR TS = (dementia)) OR TS = (mild cognitive impairment)) NOT TS = (schizophrenia)) NOT TS = (major depressive disorder)) NOT TS = (bipolar disorder)) NOT TS = (vascular disease)) NOT TS = (Parkinson) AND ((TS = (neurobehavioural manifestation)) OR TS = (mood)) OR TS = (behavioural symptom) AND ((((((((((((TS = (cognitive behavioural therapy)) OR TS = (cognitive stimulation)) OR TS = (cognitive training)) OR TS = (game training)) OR TS = (cognitive rehabilitation)) OR TS = (cognitive therapy)) OR TS = (amnesia rehabilitation)) OR TS = (amnesia therapy)) OR TS = (game)) NOT TS = (video game)) NOT TS = (mindfulness)) NOT TS = (virtual reality)) NOT TS = (exergaming)
Scopus	547	((TITLE-ABS-KEY(Alzheimer)) AND ((TITLE-ABS-KEY(neurobehavioural manifestation) OR TITLE-ABS-KEY(mood) OR TITLE-ABS-KEY(behavioural symptom))) AND ((TITLE-ABS-KEY(cognitive behavioural therapy) OR TITLE-ABS-KEY(cognitive stimulation) OR TITLE-ABS-KEY(cognitive training) OR TITLE-ABS-KEY(game training) OR TITLE-ABS-KEY(cognitive rehabilitation) OR TITLE-ABS-KEY(cognitive therapy) OR TITLE-ABS-KEY(amnesia rehabilitation) OR TITLE-ABS-KEY(amnesia therapy) OR TITLE-ABS-KEY(game) AND NOT TITLE-ABS-KEY(video game) AND NOT TITLE-ABS-KEY(mindfulness) AND NOT TITLE-ABS-KEY(virtual reality) AND NOT TITLE-ABS-KEY(exergaming))) AND PUBYEAR >2012 AND PUBYEAR <2024 AND (LIMIT-TO (SUBJAREA,"MEDI”) OR LIMIT-TO (SUBJAREA,"NEUR”) OR LIMIT-TO (SUBJAREA,"PSYC”) OR LIMIT-TO (SUBJAREA,"HEAL”) OR LIMIT-TO (SUBJAREA,"SOCI”)) AND (LIMIT-TO (DOCTYPE,"ar”)) AND (LIMIT-TO (LANGUAGE,"English”) OR LIMIT-TO (LANGUAGE,"Spanish”)) AND (LIMIT-TO (SRCTYPE,"j”)))
Embase	969	#1 MeSH descriptor: [Alzheimer Disease] 1 tree(s) exploded 5,293 #2 MeSH descriptor: [Neurocognitive Disorders] explode all trees 16,626 #3 (Alzheimer):ti,ab,kw 13,355 #4 (neurocognitive disorder):ti,ab,kw 1,251 #5 (mild cognitive impairment):ti,ab,kw 5,338 #6 (“Parkinson”):ti,ab,kw 12,301 #7 MeSH descriptor: [Neurobehavioural Manifestations] 1 tree(s) exploded 7,080 #8 MeSH descriptor: [Affect] explode all trees 5,232 #9 (mood):ti,ab,kw 25,172 #10 MeSH descriptor: [Cognitive Training] explode all trees 28 #11 (cognitive stimulation):ti,ab,kw 6,176 #12 (cognitive training):ti,ab,kw 17,215 #13 (cognitive rehabilitation):ti,ab,kw 7,966 #14 (game):ti,ab,kw 5,193 #15 (cognitive therapy):ti,ab,kw 47,266 #16 (amnesia therapy):ti,ab,kw 729 #17 (amnesia rehabilitation):ti,ab,kw 136 #18 #1 OR #2 OR #3 OR #4 OR #5 NOT #6 with Publication Year from 2013 to 2025, in Trials 16,157 #19 #7 OR #8 OR #9 with Publication Year from 2013 to 2025, in Trials 18,709 #20 #10 OR #11 OR #12 OR #13 OR #14 OR #15 OR #16 OR #17 with Publication Year from 2013 to 2025, in Trials 46,535 #21 #18 AND #19 AND #20 with Publication Year from 2013 to 2025, in Trials 969

*Filters were applied based on the publication year, type of article, and language of the article.

Studies were assessed for inclusion, and data were extracted and charted via Excel software. The data charting form was continually updated and adapted throughout the charting process. The articles were collated thematically by the setting, study population, sample size, methodology, type of intervention, and instrument used to assess NPS. The findings were summarised and presented via a descriptive–analytical approach. This consistent approach made it possible to compare findings, identify inconsistencies, and recognise gaps that required further examination.

Articles were not appraised for quality because the goal of this scoping review was to present all findings pertaining to the impact of cognitive therapies on NPS in people with MCI or dementia rather than to examine generalisability on the basis of quality/rigour, as is common in systematic reviews (SRs; Arksey and O’Malley, 2005).

## 3 Results

### 3.1 Search results

The original search strategy yielded 3,177 articles across the four databases in addition to 3 articles that were found through manual searching. Of these, 1,323 articles were excluded because they were duplicates, and another 1777 were excluded because they met one or more of the exclusion criteria mentioned above. A review of the title and abstract led to the exclusion of 8 additional articles.

**Figure 1:**
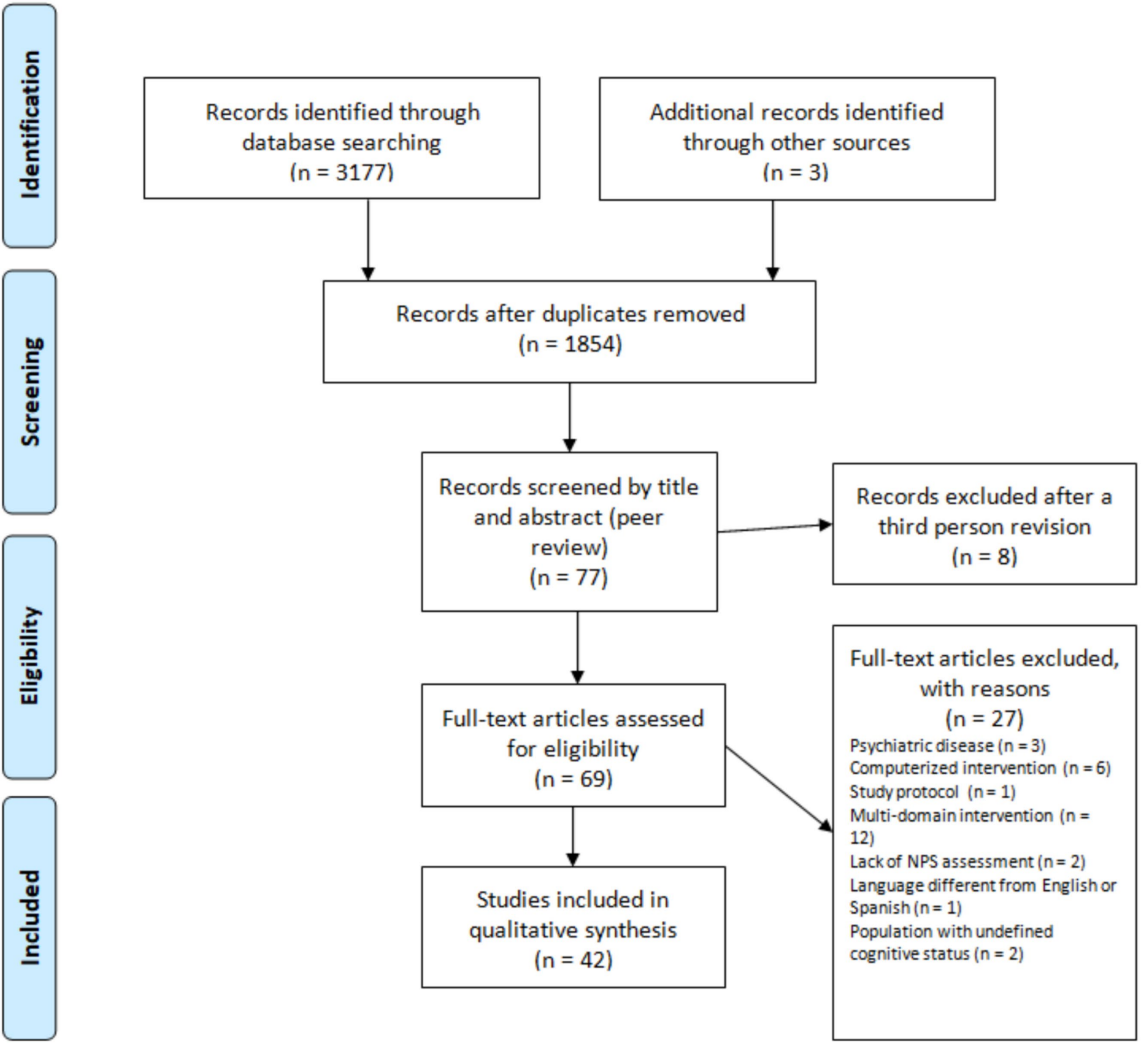
PRISMA flow diagram.

Of the remaining 69 articles, 27 were excluded on the basis of full-text screening by two reviewers with conflicts resolved by a third reviewer, yielding a total of 42 articles that were included in the review. All of these processes are detailed in Figure 1.

### 3.2 General description of the studies

The findings of each article are presented in Table 2. A summary of the most significant findings follows.

In terms of location, Europe was the main continent for this type of research with 24 of 42 articles published, including 7 from Italy and 5 from Spain. Asia followed and contributed 9 studies, whereas 6 were from America, 2 were from Australia, and only 1 was from Africa.

With respect to the methodology used, most of the articles were randomised controlled trials (RCTs; 31 of 42), which increases the quality of this scoping review. Five articles were descriptive studies, 5 were quasiexperimental studies, and 1 was a mixed methods study.

The number of sessions of the included studies ranged from 4 to 148, with an average of 20 sessions. The studies were categorised based on the duration of treatment: short-term (8 weeks or less) or long-term (greater than 8 weeks; Sherman et al., 2017). In this scoping review, only 21% of the studies reported short treatment durations for all types of interventions except CS, which was performed only for a long duration. The median number of sessions for all studies was 12.

A total of 4,089 individuals were included across the 42 studies for which quantitative analysis was conducted. The minimum sample size was 1 (for a case report), and the maximum sample size was 475 participants. The participants can be classified by the aetiology of their cognitive impairment. A total of 1,181 participants had MCI, 399 had AD, 1497 had unspecified dementia, 563 had MCI or dementia (the articles did not specify the number of participants for each condition), 179 were healthy older adults in the control group, and 270 had subjective cognitive impairment (SCI).

For the synthesis analysis, we focused on positive and significant results for NPS after a cognitive intervention. Although the majority of the articles did not use NPS as the primary outcome, all of them included at least one instrument to assess depression, anxiety, or a combination of both or used global NPS scales. Thirty-one articles assessed depressive symptoms: 18 used the Geriatric Depression Scale (GDS), 10 used the Cornell Scale for Depression in Dementia (CSDD), and the others used diverse instruments. Depression was also assessed in combination with anxiety. Nine of the 39 articles assessed this combination, most of which employed the Hospital Anxiety and Depression Scale (HADS). Anxiety as an isolated construct was assessed in 5 studies with four different instruments. Ten studies assessed NPS in general, 9 of which used the Neuropsychiatric Inventory (NPI). Two articles assessed apathy and self-efficacy.

Among the 42 articles, 26 had significant results for one or more NPS (62%), and the other 4 had positive but not significant results (10%). All of these studies included an average of 16 sessions.

### 3.3 Cognitive training

Three RCTs reported significant results for NPS after a CT intervention. Giovagnoli et al. examined a study group from Italy using a CT intervention in 50 individuals with AD for 24 sessions and reported significantly lower levels of depression and anxiety posttreatment (Giovagnoli et al., 2017). Lalanne et al. examined a study group from France using another CT intervention for individuals with AD but with a smaller sample size (Balestrieri, 2000) and lower number of sessions (Farias et al., 2009). However, these authors also reported significant results. The participants depressive symptoms improved at both posttreatment and follow-up (Lalanne et al., 2015). A study of patients with MCI from China included 24 CT sessions and reported improvements in depressive symptoms (Xue et al., 2021). Only one study utilised an individual intervention (Lalanne et al., 2015), whereas the other two studies employed group intervention (Giovagnoli et al., 2017; Xue et al., 2021).

### 3.4 Cognitive rehabilitation

Of the 6 articles that employed a CR intervention, 3 showed significant results, whereas the others yielded mixed outcomes. Specifically, one study reported reduced depressive symptoms (Tonga et al., 2021), the second study reported improvements in memory self-efficacy but not in depressive symptoms (Greenaway et al., 2013), and the third study reported deficits in abnormal motor behaviour (Brunelle-Hamann et al., 2015). Another study reported positive but not significant results for anxiety and depression (Sullivan et al., 2015). Five of the 6 interventions in these studies were conducted through individual sessions. Only Greenaway et al. used a group session-based CR intervention for MCI patients and reported significant improvements in memory self-efficacy but not in depressive symptoms at the end of the study. Moreover, this RCT included a nonactive control group, which makes it more challenging to attribute the positive effects solely to the CR intervention rather than the mere act of receiving an intervention (Greenaway et al., 2013). Despite the lack of significant results, O’Sullivan et al. reported improvements in anxiety and depression in 5 MCI participants (Sullivan et al., 2015). The most surprising result was reported by Brunelle-Hamann et al., who noted that their participants with AD displayed significantly worsened aberrant motor behaviour during the intervention (Brunelle-Hamann et al., 2015).

### 3.5 Cognitive stimulation

CS was the most commonly used intervention; 16 articles included this type, most of which were group interventions. Three studies reported significant results for depressive symptoms in patients with dementia following an individual CS intervention (Justo-Henriques et al., 2023), a mixed individual and group intervention (Cheng et al., 2024) and a group intervention (Kwon and Kim, 2025). The other four studies included patients with dementia as the target group for the CS intervention, all of which showed improved depression after the intervention (Stewart et al., 2017; Yamanaka et al., 2013; Marinho et al., 2021; Capotosto et al., 2017). However, one study reported positive but not significant effects of the intervention on anxiety and irritability (Capotosto et al., 2017). Zubatsky et al. conducted a quasiexperimental study with 251 patients with MCI or dementia in which depression also improved (Zubatsky et al., 2023), and Gómez-Soria et al. conducted an RCT with 337 individuals (healthy subjects, SCI, MCI and dementia) and reported significantly better scores for anxiety symptoms and improved (although not significantly) depressive symptoms (Gómez-Soria et al., 2023a). However, this study had a significant limitation as the control group was passive, making it susceptible to placebo bias.

### 3.6 Art therapy

Among the 8 articles that involved art therapy, 6 reported significant positive results for the intervention on NPS. All of them used art-based interventions in group sessions, while the studies that did not find significant results for mood employed individual interventions. Gómez and Gómez used an art therapy intervention with 42 AD patients in Spain and reported significant improvements in anxiety and depression and significantly lower total scores for NPS and for most of the individual items in the NPI (Gómez-Gallego and Gómez-García, 2017). Savazzi et al. reported that their art programme significantly improved the frequency and distress of NPS in patients with AD (Savazzi et al., 2020). Lin et al. reported significant improvements in anxiety and depression for 135 patients with MCI in China. However, because this RCT employed a passive control group, the results should be interpreted with caution (Lin et al., 2022). Xue et al. also reported positive and significant results for depression among MCI patients in China who received art therapy (Xue et al., 2023). Masika et al.’s study in Tanzania reported that an art therapy intervention with 127 MCI patients was significantly effective for improving depressive symptoms (Masika et al., 2021). Mangiacotti et al. developed an individual art therapy intervention for people with dementia that significantly improved depression; however, no significant changes were found in any of the items on the NPI (Mangiacotti et al., 2024).

### 3.7 Reminiscence therapy

Five out of the 7 articles on reminiscence therapy reported significant improvements in mood (Tonga et al., 2021; Villasán Rueda et al., 2021; Villasán-Rueda et al., 2023; Lopes et al., 2016; Lök et al., 2019). A group from Spain conducted two similar RCTs with healthy subjects, MCI patients and AD patients who received reminiscence therapy in group sessions and reported significant improvements in depression (Villasán Rueda et al., 2021; Villasán-Rueda et al., 2023). Another RCT also used reminiscence therapy with AD patients only and reported similar results (Lök et al., 2019). The other significant studies were individual approaches (Tonga et al., 2021; Lopes et al., 2016). Lopes et al. conducted a quasiexperimental study of patients with MCI and dementia and reported significant positive results for both depression and anxiety (Lopes et al., 2016).

### 3.8 Psycho-behavioural therapy

Only two studies reported on psycho-behavioural cognitive therapy programmes. Lin et al. conducted a programme with 171 patients with MCI using a mixed-methods study in China. The intervention included 13 group sessions. The authors reported significant improvements in overall NPS, particularly apathy, anxiety and depression, as measured by the Geriatric Depression Scale Short Form, Apathy Evaluation Scale and Kessler Psychological Distress Scale (Lin et al., 2023). Another psycho-behavioural study conducted by Rotenberg et al. in Canada involved 264 individuals with subjective cognitive decline or MCI who participated in a 10-session intervention that combined individual and group formats; however, no significant changes in mood were found (Rotenberg et al., 2024). One possible explanation for these differing outcomes may lie in the nature of the interventions. Lin et al. implemented an empowerment-based educational programme for stress adaptation, cognitive coping and knowledge enhancement. Rotenberg et al. developed a meta-cognitive intervention that focused on strategy acquisition and application with the aim of improving the participants’ performance in individual daily activities they deemed important.

### 3.9 Combined cognitive therapies

Only 2 of 42 articles employed combined cognitive therapies. Tonga et al. combined CR and reminiscence therapy in individual sessions for 198 participants with MCI or dementia. These authors reported significant and positive improvements in depression at posttreatment and follow-up but no improvements in global NPS scales (Tonga et al., 2021). Carter et al. conducted a descriptive study that involved a case report of a person with dementia who participated in a multimodal intervention composed of CT, art therapy and reminiscence therapy but found no significant difference in mood (Carter et al., 2019).

## 4 Discussion

This scoping review was designed to collect valuable information about how nonpharmacological interventions, particularly cognitive therapies, impact NPS in people with cognitive disorders due to MCI or dementia.

### 4.1 Intervention efficacy

After analysing the results, we observed that there was no clear pattern of the efficacy of cognitive interventions on NPS. Although there were many RCTs and studies with large sample sizes, none of these studies appeared to be superior to the others. However, we observed some tendencies. A comparison of the effects of the different types of cognitive interventions revealed that art therapies and reminiscence therapy were the most promising: 75% of the art therapy interventions and 71% of the reminiscence therapy interventions were effective at decreasing NPS, compared with 56% of interventions using cognitive stimulation (CS) and cognitive rehabilitation (CR) and 43% of interventions using cognitive training (CT). Our review revealed that psychobehavioural programmes were 50% effective; however, only two studies used this type of intervention, so we cannot fully determine its efficacy in treating depression, anxiety and NPS.

Art-based interventions seem to be an extended therapy for several pathologies and conditions and are used frequently in health care institutions and community projects (Vaartio-Rajalin et al., 2021). Art therapy is used effectively in patients with dementia to engage patients in relationships with others and to increase their motivation, self-expression and self-esteem (Stewart, 2004). In fact, our findings suggest that art therapy in the MCI population improves depressive and anxious symptoms when conducted in a group setting (Lin et al., 2022; Xue et al., 2023; Masika et al., 2022). It also improves NPS in populations with AD, as assessed by the NPI (Gómez-Gallego and Gómez-García, 2017; Savazzi et al., 2020). Furthermore, studies have demonstrated that brain changes due to art therapy are associated with improvements in cognitive processes such as planning, creativity, verbal expression, decision-making, cognitive control and abstract thinking (De Pisapia et al., 2016; Bolwerk et al., 2014; Lusebrink, 2004). Therefore, it is reasonable that art-based interventions are good options for treating cognitive impairment and its implications, such as cognitive decline, as well as psychological and behavioural symptoms such as NPS. A SR of SRs of non-pharmacological interventions concluded that among sensory stimulation interventions, music therapy was the only effective approach for reducing agitation and aggressive behaviour, although other interventions, including music therapy, were also effective in improving anxiety (Abraha et al., 2017).

With respect to reminiscence therapy, many experimental and review publications on patients with MCI or dementia reported better cognitive functioning and quality of life and a reduction in depressive symptoms following reminiscence therapy (Lök et al., 2019; González-Arévalo, 2015; Moon and Park, 2020; O’Philbin et al., 2018; Park et al., 2019; Abu Khait et al., 2021; Ching-Teng et al., 2020; Van Bogaert et al., 2016), as did our review, which suggests a positive effect of reminiscence therapy on reducing depressive symptoms. Reminiscence therapy is an intervention in which participants discuss important events and past experiences using photographs, objects or music to evoke memories (Spector et al., 2000). A possible explanation for its efficacy may be that reminiscence therapy is a less anxiety-producing and more attractive strategy in which the participants communicate and connect with their past (Caddell and Clare, 2010; Dempsey et al., 2014).

Our findings for CT are somewhat controversial. CT is a type of cognitive intervention that focuses on exercising selected cognitive processes to optimise functional capacity related to the trained activity in addition to improving related cognitive processes (García-Ribas et al., 2023). In our review, we observed that a variety of methodologies were employed, making it difficult to combine the results; this finding is consistent with the mixed findings for the benefits of cognitive training (Sala and Gobet, 2019). Some studies have reported benefits from cognitive training (Reijnders et al., 2013; Sanz Simon et al., 2012); however, others found no remarkable advantages (Gates et al., 2011; Belleville, 2008). There is a lack of methodological consistency among the studies, which would contribute to determining a clear connection between CT and changes in NPS. Because one of our eligibility criteria required face-to-face interventions, several studies that employed CT approaches could not be included in this scoping review as they involved computerised interventions. There is a growing body of scientific literature on the use of computerised or virtual interventions for MCI or dementia (Hill et al., 2017; Beishon et al., 2022). It would be valuable to explore how these interventions impact not only cognitive outcomes but also NPS. While many studies have conducted multimodal interventions (i.e., interventions that combine psychological, physical, cognitive and social strategies; Li et al., 2021; Baglio et al., 2015; Ekman et al., 2020; Koskas et al., 2022; Luttenberger et al., 2019; Quail et al., 2020; Takeda et al., 2020), we found only two studies that combined several cognitive therapies and evaluated NPS (Tonga et al., 2021; Carter et al., 2019). Although these studies revealed improvements in NPS, especially depression, further research is needed to observe the trend of the effectiveness of combined cognitive interventions for decreasing NPS. However, it appears that future approaches to cognitive impairment may involve multidomain interventions that combine various cognitive therapies or integrate cognitive interventions with physical treatments, which have shown positive effects on NPS (Baglio et al., 2015; Yorozuya et al., 2024; Bartels et al., 2022; Han et al., 2017). Furthermore, we sought to identify patterns based on the type of cognitive impairment. We reviewed studies with positive outcomes of improved mood and overall NPS in which the interventions focused specifically on MCI or dementia, including AD or unspecified dementias, but excluded those that combined different cognitive disorders to accurately assess the true effect. Again, the pattern remained unclear. CS appeared to be the most commonly used intervention for dementia (accounting for 53.25% of dementia interventions). In contrast, only 25% of studies involving patients with MCI applied this type of intervention, with art-based interventions being the most common and effective approach for improving NPS in this group. Of the 26 studies conducted on dementia, 16 reported positive outcomes in NPS, representing 61.5% of the studies in this group. In contrast, 8 out of 15 MCI studies (53.3%) reported positive effects. These results may suggest that cognitive interventions are more effective for improving NPS in patients with dementia than in patients with MCI.

### 4.2 Target symptoms

A recent study showed that caregiver time for patients with dementia is a significant unpaid informal cost that represents between 31 and 49% of the total cost of dementia care in the United States of America (USA; Hurd et al., 2013). This informal cost is associated not only with disease severity but also with the presence of NPS (Rattinger et al., 2015). A more recent study in the USA concluded that strategies that decrease caregiver burden, such as therapies that impact the NPS of patients with dementia, are important not only for these patients’ well-being but also at an economic level (Rattinger et al., 2019).

We found that depression is the most frequently targeted symptom, either alone or in combination with anxiety or other NPS, consistent with another recent scoping review that reported that depression is the most prevalent NPS in prodromal and late-stage AD (Ferreira et al., 2023). Depression is also the most common symptom in patients with MCI (83%; Martin and Velayudhan, 2020) and is considered a risk factor for dementia in both patients with MCI and cognitively normal older adults (Rosenberg et al., 2018). However, depression can also be a cause of cognitive impairment and is associated with MCI; cognitively normal older adults with depression have a 1.5-fold increased risk of progression to MCI (Hu et al., 2020), although the symptoms may be reversed (Ismail et al., 2017).

In this scoping review, only three articles did not explicitly assess depressive symptoms. These articles measured depressive symptoms implicitly using the NPI instrument; thus, depression was the main symptom studied in people with cognitive impairment after cognitive intervention. Overall, 25 studies reported beneficial effects of cognitive interventions on depressive symptoms (Giovagnoli et al., 2017; Xue et al., 2021; Greenaway et al., 2013; Sullivan et al., 2015; Justo-Henriques et al., 2023; Cheng et al., 2024; Kwon and Kim, 2025; Yamanaka et al., 2013; Marinho et al., 2021; Capotosto et al., 2017; Gómez-Gallego and Gómez-García, 2017; Xue et al., 2023; Mangiacotti et al., 2024; Villasán Rueda et al., 2021; Villasán-Rueda et al., 2023; Lopes et al., 2016; Lin et al., 2023; Bademli et al., 2018; Carbone et al., 2021; Gómez-Soria et al., 2023c; Lalanne et al., 2015; Lin and Li, 2022; Tonga et al., 2021; Zubatsky et al., 2023). The other frequently studied NPS in the collected studies was anxiety, which was reported as the second most prevalent symptom in a recent review (Ferreira et al., 2023). This high prevalence is linked to cognitive decline in older adults (Palmer et al., 2007; Deluca et al., 2005; Gallacher et al., 2009; Sinoff and Werner, 2003; Andreescu et al., 2014), so some studies consider anxiety a risk factor for dementia (Pietrzak et al., 2012). Nevertheless, other studies have concluded that anxiety may be related to psychiatric illness, and conflicting results exist that do not allow for the generalisation of anxiety as a dementia risk (Ismail et al., 2017).

Although there is evidence of the efficacy of pharmacological treatment in decreasing anxiety symptoms, many older adults prefer nonpharmacological interventions. These interventions seem to work when they are well designed according to the needs, expectations and cultural background of older patients with anxiety (Andreescu and Lee, 2020). This may explain the results of our scoping review, in which 9 studies reported positive impacts of cognitive therapy on anxiety symptoms. Only 5 other studies assessed anxiety, and these studies did not find positive results. Despite these mixed results and the association between anxiety and cognitive disorders, further research on this topic is clearly needed.

### 4.3 Assessment of neuropsychiatric symptoms

One of the objectives of this review was to examine the instruments that are most frequently employed to assess symptoms. We observed that for NPS, the most commonly used instruments were the Neuropsychiatric Inventory (NPI) and the Geriatric Depression Scale (GDS) for depression. These findings are consistent with those of a recent SR (Ferreira et al., 2023).

Although most of the articles analysed in this scoping review included cognitive domains as a primary outcome (Giovagnoli et al., 2017; Xue et al., 2021; Justo-Henriques et al., 2023; Cheng et al., 2024; Kwon and Kim, 2025; Stewart et al., 2017; Yamanaka et al., 2013; Marinho et al., 2021; Capotosto et al., 2017; Savazzi et al., 2020; Lin et al., 2022; Xue et al., 2023; Masika et al., 2021; Mangiacotti et al., 2024; Villasán Rueda et al., 2021; Villasán-Rueda et al., 2023; Lopes et al., 2016; Bademli et al., 2018; Carbone et al., 2021; Lalanne et al., 2015; Lin and Li, 2022; Zubatsky et al., 2023; Gómez-Soria et al., 2022; Gómez-Soria et al., 2023a; Carbone et al., 2022; Middelstadt et al., 2016; Greenaway et al., 2013; Vidovich et al., 2015; Kelly et al., 2019; Bergamaschi et al., 2013; Cavallo et al., 2013; Gómez-Gallego and Gómez-García, 2017; Tsai et al., 2019; Justo-Henriques et al., 2022), some studies included other constructs as the main outcome measure (Tonga et al., 2021; Brunelle-Hamann et al., 2015; Sullivan et al., 2015; Lin et al., 2023; Rotenberg et al., 2024; Carter et al., 2019; Clare et al., 2019b; Regan et al., 2017), mostly NPS. However, the number of studies that targeted NPS in general, not just depression and anxiety, was surprising. Given the considerable impact of NPS in the daily life of people with cognitive impairment (Ferreira et al., 2023; Scaricamazza et al., 2019; Zhao et al., 2016; Liew, 2021; Mallo et al., 2020), there continues to be a gap in knowledge of how nonpharmacological interventions, specifically cognitive therapies, impact overall NPS.

### 4.4 Intervention characteristics

Twelve studies did not find significant positive results in terms of improvement in NPS. Eight of these 12 studies employed individual approaches (Rotenberg et al., 2024; Carter et al., 2019; Carbone et al., 2021; Cavallo et al., 2013; Justo-Henriques et al., 2022; Regan et al., 2017; Clare et al., 2019a; Kelly et al., 2019), which represented 67% of the studies. In contrast, among the 30 articles with significant positive results for NPS, only 8 employed individual sessions (Lalanne et al., 2015; Tonga et al., 2021; Brunelle-Hamann et al., 2015; Sullivan et al., 2015; Justo-Henriques et al., 2023; Cheng et al., 2024; Mangiacotti et al., 2024; Lopes et al., 2016), a total of 27% of the studies. Moreover, the only study that showed significantly worsened NPS scores involved an individual intervention (Brunelle-Hamann et al., 2015). A theory derived from Zubatsky et al. is that socialisation helps to increase the overall perceptions of the group and individuals’ self-esteem (Zubatsky et al., 2023), which may be consistent with the fact that group interventions seem to be more effective than individual interventions in enhancing mood.

The length of the studies (i.e., the duration of intervention (number of hours)) did not seem to have a large influence on the impact of the cognitive intervention on NPS. These findings are consistent with those of an SR in individuals with MCI (Sherman et al., 2017) and dementia (Carrion et al., 2018). The studies that yielded significant results used cognitive interventions that ranged from 5 sessions to 47 sessions. Very long interventions (more than 100 sessions) may not be effective for NPS, but further research is needed to determine whether the effect of the duration of the intervention can be isolated from other confounding variables.

### 4.5 Limitations and strengths of the study: knowledge gaps and opportunities for further research

Although scoping reviews have many advantages, such as being a precursor to an SR, they also have limitations. The main limitation is that they are not replicable because there is no established protocol with specific search criteria for collecting information, so scoping reviews lack methodological rigour compared with SRs (Lopez-Cortes et al., 2022). Another limitation is that most studies included in this review have a possible risk of bias due to the lack of double-blinded randomisation. In addition, many of these studies did not use detailed randomisation procedures and were especially heterogeneous (e.g., duration of sessions, length of treatment, group or individual approaches, number of participants, aetiology of the cognitive impairment). These possible biases compromise the generalisability of the results and make it difficult to draw conclusions about the best cognitive intervention to improve NPS and, concurrently, caregivers’ well-being. Another limitation is that we did not consider sex differences in NPS because the studies included in this review did not mention this information.

As observed in previous SRs (McDermott et al., 2013; Subramaniam and Woods, 2012; Carrion et al., 2013), cognitive therapies exhibit significant methodological heterogeneity. Perhaps the most limiting factor is the large differences in the characteristics of the interventions even within the same type of cognitive intervention, which makes it difficult to conduct meta-analyses. This is the primary reason why the present study is a scoping review, as mentioned in the methodology section, as this type of review is suitable for analysing articles with different study designs. Despite the inability to conduct a meta-analysis and although cognitive therapies were previously explored through a scoping review with positive findings for NPS, we believe it would be appropriate to conduct an SR to strengthen the methodological analysis.

**Table 2:** Studies included in the scoping review.

Author and year	Country	Type of study	Sample population	Type of intervention	Cg intervention	Intervention characteristics	Assessment	Outcomes
Bademli et al. (2018)	Turkey	RCT	*n* = 60 AD	Reminiscence therapy	Passive	8 group sessions Once a week for 8 weeks	CSDD	Significant improvement in depressive symptoms in experimental group compared to CG (*p* 0.001)
Bergamaschi et al. (2013)	Italy	RCT	*n* = 32 AD	CT	Active (nonspecific cognitive exercises)	100 group sessions 51-month cycles (one cycle: 5 days a week) with a break of 4 weeks in between each cycle	CSDD	No significant improvements in depressive symptoms
Brunelle-Hamann et al. (2015)	Canada	RCT	*N* = 15 AD	CR	Passive	8 individual sessions Twice a week for 4 weeks	NPI	AMB significantly worsened in AD participants during the intervention (*p* 0.03)
Capotosto et al. (2017)	Italy	RCT	*N* = 39 Dementia	CS	Active (alternative general activities)	14 group sessions Twice a week for 7 weeks	CSDD NPI	Significant decreases in depression levels in experimental group (*p* 0.001). No significant results in NPI, but lower scores for anxiety and irritability in experimental group
Carbone et al. (2021)	Italy	RCT	*N* = 235 Dementia	CS	Active (alternative educational activities)	14 group sessions Twice a week for 7 weeks	CSDD NPI	Slight decrease (not significant) in depression symptoms Neuropsychiatric symptoms did not increase (they did in the CG)
Carbone et al. (2022)	Italy	RCT	*N* = 123 Dementia	CS	Active (TAU)	6 individual sessions (once a week) + 14 group sessions (twice a week for 7 weeks)	CSDD NPI	Less frequent and less severe behavioural and neuropsychiatric symptoms at T1, but not maintained at T2
Cavallo et al. (2013)	Italy	Quasiexperimental study	*N* = 11 AD	CT	Active (same treatment as experimental group)	4 individual sessions	HADS	No significant differences in mood
Cheng et al. (2024)	China	RCT	*N* = 76 MCI	CS	Active (TAU)	24 combined individual and group sessions 3 weekly sessions over 8 weeks	GDS	Significant improvement in depressive symptoms in experimental group (*p* = 0.01)
Clare et al. (2019a)	UK	RCT	*N* = 475 Dementia	CR	Active (TAU)	14 individual sessions 10 weekly sessions over 3 months and 4 maintenance sessions over 6 months	HADS	No significant differences in mood
Giovagnoli et al. (2017)	Italy	RCT	*N* = 50 AD	CT	Active (music therapy and neuroeducation)	24 group sessions Twice a week for 12 weeks	BDI STAI Y-1 and STAI Y-2	Significantly lower depression levels in all groups at T1 (*p* 0.013); all groups showed significant improvements in trait anxiety at T1 (*p* 0.028)
Gómez-Gallego and Gómez-García (2017)	Spain	Descriptive study	*N* = 42 AD	Art therapy	–	12 group sessions Twice per week for 6 weeks	HADS NPI	Significant improvements in anxiety and depression (*p* < 0.001), significantly lower total NPI score (*p* < 0.001) and lower scores on most items (delirium *p* 0.015, hallucinations *p* 0.026, agitation 0.010, anxiety 0.002, apathy 0.034, irritability 0.025)
Gómez-Soria et al. (2021)	Spain	RCT	*N* = 50 MCI	CS	Active (TAU)	10 group sessions Once a week	GADS GDS	Improvement trend (not significant) for anxiety and depression at T1 and T2
Gómez-Soria et al. (2023a); Gómez-Soria et al. (2023b); Gómez-Soria et al. (2023c)	Spain	RCT	*N* = 337 101 Healthy subjects 100 SCI 108 MCI 28 Dementia	CS	Passive	10 group sessions Once a week	GADS GDS	Significant differences in anxiety at T1 in SCI (*p* 0.006) and in MCI (*p* 0.005); improvement trend (not significant) in depression
Greenaway et al. (2013)	USA	RCT	*N* = 40 MCI	CR	Passive	12 group sessions Twice a week for 6 weeks	SEM-MCI	Significant improvements in memory self-efficacy in experimental group (*p* < 0.01) and between groups (*p* 0.02)
Justo-Henriques et al. (2022)	Portugal	RCT	*N* = 251 Dementia	Reminiscence therapy	Active (TAU)	26 individual sessions Twice a week for 13 weeks	GDS	No significant differences in mood
Justo-Henriques et al. (2023)	Portugal	RCT	*N* = 59 Dementia	CS	Active (TAU)	47 individual sessions Once a week	GDS	Experimental group significantly improved depressive symptoms from intra-intervention to endpoint (*p* < 0.001) and compared to CG at endpoint (*p* 0.001). In contrast, CG worsened at the endpoint (*p* 0.031)
Kelly et al. (2019)	Ireland	Descriptive study	*N* = 3 AD	Art therapy	–	8 individual sessions Once a week	HADS	No significant differences in mood
Kwon and Kim (2025)	Republic of Korea	RCT	*N* = 60 AD	CS (experimental group)	Active (cognitive workbook)	20 group sessions 5 times a week over 4 weeks	GDS	Significant intragroup differences (both experimental and CG *p* < 0.001) and comparing experimental group and CG with greater improvement in experimental group (*p* < 0.001)
Lalanne et al. (2015)	France	RCT	*N* = 33 AD	CT	Active (another CT programme)	6 individual sessions Once a week	GDS	Significant improvements in experimental group for mood at T1 (*p* < 0.001) and T2 (*p* < 0.001) and in CG at T2 (*p* 0.003); experimental group showed significant improvement compared to CG at T2 (*p* < 0.01)
Lin and Li (2022)	Taiwan	RCT	*N* = 30 Dementia	CS	3 groups: olfactory simulation group, board game group and CG TAU	24 group sessions Twice a week for 12 weeks	GDS	Improvement trend (not significant) in all groups
Lin et al. (2022)	China	RCT	*N* = 135 MCI	Art therapy	Active CG (puzzle games) and passive CG	24 group sessions Once a week	SAS GDS	Significant differences between the intervention group and the passive CG in depression (*p* 0.05) and anxiety (*p* 0.009)
Lin et al. (2023)	China	Mixed method study	*N* = 171 MCI	Psycho-behavioural programme	Active (health education programme)	13 group sessions Once a week	Mild Behavioural Impairment Checklist GDS Apathy Evaluation Scale Kessler Psychological Distress Scale	Significant improvements in overall neuropsychiatric symptoms in experimental group at T1 (*p* < 0.001) and T2 (P.006) and between groups at T1 (*p* 0.044) and at T2 (*p* 0.018); apathy in experimental group at T1 (*p* < 0.001) and T2 (*p* < 0.001) and between groups at T1 (*p* 0.018) and T2 (*p* < 0.001); anxiety in experimental group at T1 (*p* 0.003) and T2 (*p* < 0.001) and between groups at T1 (*p* 0.009) and at T2 0.023); and depression in experimental group at T1 (*p* 0.004) and T2 (*p* < 0.001) and between groups at T2 (*p* < 0.001)
Lopes et al. (2016)	Portugal	Quasiexperimental study	*N* = 41 MCI or dementia	Reminiscence therapy	Active (TAU)	5 individual sessions Once a week	CSDD GDS GAI	Experimental group showed significant decrease in depression observed in GDS (*p* < 0.001) and in anxiety symptoms (*p* < 0.01) compared to CG
Mangiacotti et al. (2024)	UK	RCT	*N* = 42 Dementia	Art therapy	Active (storytelling)	16 individual sessions Once a week	CSDD NPI	Only the intervention group showed significant improvement in depression with CSDD (*p* 0.001); no significant changes in NPI
Marinho et al. (2021)	Brasil	RCT	*N* = 47 Dementia	CS	Active (TAU)	14 group sessions Twice a week for 7 weeks	CSDD	The intervention group showed significantly lower scores after the intervention (*p* 0.003), while the CG had an increase in depressive symptoms (*p* 0.022)
Masika et al. (2022)	Tanzania	RCT	*N* = 127 MCI	Art therapy	Active (health education sessions)	12 group sessions Twice a week for 6 weeks	GDS	Experimental group (*p* < 0.001) and CG (*p* < 0.001) showed significant reduction in depression, but experimental group showed a significantly greater reduction (*p* < 0.001)
Middelstadt et al. (2016)	Germany	RCT	*N* = 71 Dementia	CS	Active (TAU)	16 group sessions Twice a week for 8 weeks	NPI	No significant differences in mood
Sullivan et al. (2015)	Ireland	Descriptive study	*N* = 5 MCI	CR	–	6 to 8 individual sessions Once a week	HADS	Improvement trend (not significant) in anxiety and depression
Regan et al. (2017)	Australia	RCT	*N* = 40 MCI or dementia	CR	Active (TAU)	4 individual sessions Once a week	HADS	No significant differences in mood
Rotenberg et al. (2024)	Canada	RCT	*N* = 264 170 SCI 62 MCI 32 Nonspecified	Psycho-behavioural programme	Active (brain education)	10 sessions Once a week 8 group sessions and 2 individual sessions	GDS GAI	No significant differences in mood
Savazzi et al. (2020)	Italy	Quasiexperimental study	*N* = 20 AD	Art therapy	Active (TAU)	14 group sessions Twice a week for 7 weeks	NPI	Behavioural symptoms showed significant improvement in frequency (*p* < 0.005) and in distress (*p* < 0.001) in experimental group compared to CG
Stewart et al. (2017)	USA	Descriptive study	*N* = 40 Dementia	CS	–	14 group sessions Twice a week for 7 weeks	CSDD	Significant decrease in depressive symptoms (*p* 0.002)
Tonga et al. (2021)	Norway	RCT	*N* = 198 MCI or dementia	CR Reminiscence therapy	Active (TAU)	11 individual sessions Once a week	MADRS NPI	Depressive symptoms were significantly more reduced in the experimental group compared to CG (*p* < 0.001); no significant differences were found in neuropsychiatric symptoms
Tsai et al. (2019)	Taiwan	Quasi experimental study	*N* = 25 MCI	CS	Active (TAU)	14 group sessions Once a week	HADS	No significant differences in mood
Vidovich et al. (2015)	Australia	RCT	*N* = 160 MCI	CT	Active (nonspecific educational programme)	10 group sessions Twice a week for 5 weeks	PHQ-9	No significant differences in mood
Villasán Rueda et al. (2021)	Spain	RCT	*N* = 77 27 Healthy subjects 24 MCI 26 AD	Reminiscence therapy	Active (TAU)	12 group sessions Twice a week for 2 months	LSI-A GDS	Healthy subjects showed improved depression in both groups (*p* < 0.001), but the AD group improved only in the experimental group (*p* 0.006); no effects on LSI-A scores
Villasán-Rueda et al. (2023)	Spain	RCT	*N* = 144 (Mexico and Spain) 51 Healthy subjects 46 MCI 47 AD	Reminiscence therapy	Active (CS)	12 group sessions Twice a week for 6 weeks	GDS	AD patients in Spain did not show significant results, although Mexican patients in experimental group did (*p* < 0.001); for patients with MCI, Mexican sample showed statistical significance (*p* 0.006)
Xue et al. (2021)	China	RCT	*N* = 72 MCI	CT	Active (TAU)	24 group sessions Three times per week for 8 weeks	GDS	Significant improvements in depressive symptoms in experimental group (*p* < 0.001) and compared to CG (*p* < 0.001)
Xue et al. (2023)	China	RCT	*N* = 80 MCI	Art therapy	Active (TAU)	32 group sessions 4 times a week for 8 weeks	GDS	Significant improvements in depressive symptoms in experimental group (*p* < 0.001) and compared to CG (*p* < 0.001)
Yamanaka et al. (2013)	Japan	RCT	*N* = 56 Dementia	CS	Active (TAU)	14 group sessions Twice a week for 7 weeks	Face scale	Significant improvements in depressive symptoms in experimental group (*p* 0.009) and in CG (*p* 0.017)
Zubatsky et al. (2023)	USA	Quasiexperimental study	*N* = 252 MCI or dementia	CS	–	14 group sessions Twice a week for 7 weeks	CSDD	Improvements in depressive symptoms in both groups, but significant differences were observed only in patients who lived in the community compared with the residential group (*p* 0.018)

*AD, Alzheimer’s disease; AMB, aberrant motor behaviour; BDI, Beck Depression Inventory; CG, control group; CR, cognitive rehabilitation; CS, cognitive stimulation; CSDD, Cornell Scale for Depression in Dementia; CT, cognitive training; GADS, Goldberg Anxiety Subscale and Yesavage Geriatric Depression Scale; GAI, Geriatric Anxiety Inventory; GDS, Geriatric Depression Scale; HADS, Hospital Anxiety and Depression Scale; LSI-A, Life Satisfaction Index-Adults; MADRS, Montgomery–Åsberg Depression Rating Scale; MCI, mild cognitive impairment; NPI, Neuropsychiatric Inventory; PHQ-9, Patient Health Questionnaire-Nine Item; PWBS, Psychological Well-being Scale for the Elderly; SAS, Zung Self-Rating Anxiety Scale; SCI, subjective cognitive impairment; SEm-MCI, Self-Efficacy in MCI Scale; STAI Y-1 and STAI Y-2, State Trait Anxiety Inventory; T1, postintervention assessment; T2, follow-up assessment; TAU, treatment-as-usual.

The management of NPS, or the behavioural and psychological symptoms of dementia (BPSD), is widely agreed upon. The approach begins by identifying the underlying causes of the symptoms, which in some cases may be addressed with simple solutions, such as treating medical conditions or modifying the person’s immediate environment. If no improvement is observed, health care professionals typically recommend prioritising nonpharmacological interventions over medication, except in extreme cases such as psychosis or aggressive behaviour (Kales et al., 2019). Although nonpharmacological approaches have been shown to be effective in improving NPS, due to their significant impact on NPS measures and the absence of adverse events, these interventions remain poorly defined (Dyer et al., 2018). Cognitive therapies require further research to establish high-quality evidence for their treatment of NPS. While many studies have conducted multimodal interventions (i.e., interventions that combine psychological, physical, cognitive and social strategies; Li et al., 2021; Baglio et al., 2015; Ekman et al., 2020; Koskas et al., 2022; Luttenberger et al., 2019; Quail et al., 2020; Takeda et al., 2020), we found only two studies that combined several cognitive therapies and evaluated NPS (Tonga et al., 2021; Carter et al., 2019). Although these studies revealed improvements in NPS, especially depression, further research is needed to observe the trend of effectiveness of combined cognitive interventions for decreasing NPS. It appears that future approaches to cognitive impairment may involve multidomain interventions that combine various cognitive therapies or integrate cognitive interventions with physical treatments, which have shown positive effects for NPS (Baglio et al., 2015; Yorozuya et al., 2024; Bartels et al., 2022; Han et al., 2017).

Due to the variety of interventions (which were divided the interventions into six different categories: CT, CR, CS, reminiscence therapy, art therapy and psycho-behavioural programmes), few studies have been conducted on each intervention. It is possible that we could focus on publications prior to 2013 to increase the number of studies; however, we faced difficulty because, in general, there was no explicit conceptualisation of types of cognitive interventions.

A knowledge gap highlighted above is the lack of multimodal interventions specifically focused on NPS measures because the primary outcomes of cognitive interventions often emphasise cognitive metrics. It is important to stress that NPS are highly disruptive symptoms for individuals with cognitive impairment. Given the potential for cognitive interventions to positively affect these symptoms, we strongly encourage the inclusion of NPS measures in assessment protocols. This recommendation is valuable not only for fully cognitive multimodal interventions but also for other types of cognitive interventions, such as CT, CS, CR, art therapy, reminiscence therapy, and multimodal interventions that combine physical and cognitive activities.

An important area for future research may be psychobehavioural interventions. These interventions represented only two studies in our sample. One of them demonstrated significant positive effects on overall NPS, apathy, anxiety, and depression as well as subjective memory complaints and global cognition (Lin et al., 2023). These findings suggest that psychobehavioural interventions may be a promising approach that warrants further investigation to evaluate these outcomes more comprehensively.

In addition to the knowledge gaps mentioned, such as the lack of methodological equivalence across studies, the difficulties of developing an SR and the limited focus on overall NPS rather than just depression, several other points can be noted. First, there is no clear distinction between the methodologies of interventions that target patients with MCI and those that target patients with dementia. According to a previous review (Regier et al., 2017), cognitive treatments are more commonly prescribed for individuals with mild cognitive impairment or early-stage dementia than for those with moderate or severe stages. In contrast, art therapy shows more variation: activities such as art crafts and general cognitive exercises, which are related to sensory and manipulative tasks, are used more frequently in the early stages of dementia. These are primarily directed at individuals with mild or severe dementia, whereas music therapy is more commonly prescribed for individuals with moderate dementia. Furthermore, reminiscence therapy is typically intended for individuals with mild impairment (Regier et al., 2017). However, our findings differ. Among studies that targeted MCI, approximately 13% applied reminiscence therapy, whereas a slightly greater percentage (16%) used reminiscence therapy for patients with dementia. In contrast, art therapy was applied more frequently among the population with MCI (20%) than for patients with dementia (16%). Additionally, our study revealed that cognitive activities (CS, CT and CR) were applied equally in the groups with MCI (approximately 67%) and dementia (68%). Therefore, we were unable to confirm the findings of the previous review. Given these mixed results, this area warrants further investigation. In addition to the type of interventions, we sought to determine whether MCI interventions are more effective in improving mood and NPS than those that target individuals with dementia. Surprisingly, our results revealed that 53.3% of MCI interventions yielded significant improvements in NPS, whereas 64% of dementia interventions also demonstrated positive effects on NPS. Further research is needed to validate this observed trend.

Despite the limitations mentioned, this scoping review has several strengths. To our knowledge, this is the only scoping review on cognitive interventions that impact the NPS of people with cognitive impairment. This initial review offers a wider look at NPS beyond the pharmacological treatments that are more familiar to clinical professionals (Gerlach and Kales, 2020). Given the excellent results of cognitive interventions for NPS, there is a need for more scientific literature to enhance the evidence and specify more accurate treatments.

In conclusion, we can affirm that there is no clear pattern of effectiveness of cognitive interventions on NPS since all types of therapy seem to support improvements in NPS, especially depression. In particular, art therapy and reminiscence therapy stand out. Studies that investigate NPS globally and specific NPS beyond depression are lacking, and further research is needed.
